# How do babies feel pain?

**DOI:** 10.7554/eLife.07552

**Published:** 2015-04-27

**Authors:** Manon Ranger, Ruth E Grunau

**Affiliations:** Department of Pediatrics, University of British Columbia, Vancouver, Canada and Developmental Neurosciences and Child Health, Child and Family Research Institute, Vancouver, Canada; Department of Pediatrics, University of British Columbia, Vancouver, Canada and Developmental Neurosciences and Child Health, Child and Family Research Institute, Vancouver, Canadargrunau@cw.bc.ca

**Keywords:** pain, fMRI, infant, development, human

## Abstract

Functional MRI studies suggest that healthy full-term newborn babies experience some aspects of pain in a similar way to adults.

**Related research article** Goksan S, Hartley C, Emery F, Cockrill N, Poorun R, Moultrie F, Rogers R, Campbell J, Sanders M, Adams E, Clare S, Jenkinson M, Tracey I, Slater R. 2015. fMRI reveals neural activity overlap between adult and infant pain. *eLife* **4**. e06356. doi: 10.7554/eLife.06356

**Image** Imaging brain activity reveals the regions of the brain that are associated with pain

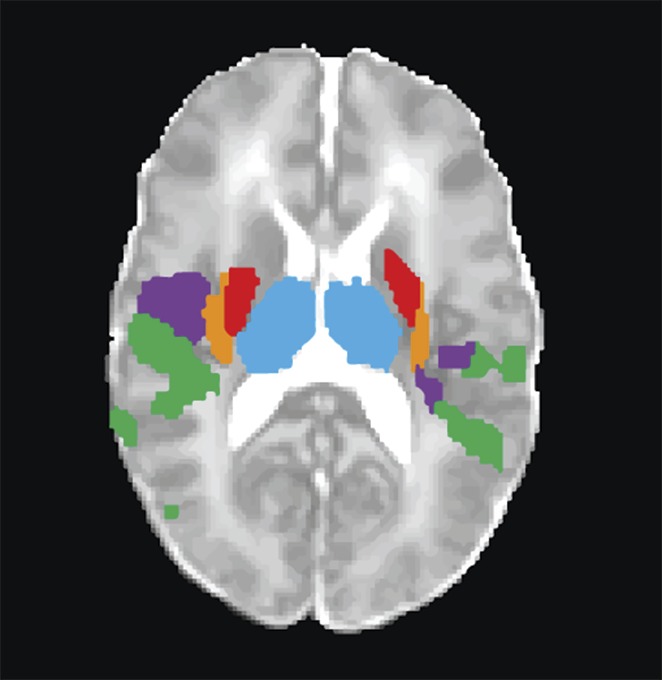

Adults and older children are able to tell someone when they are in pain. But what about newborn babies who have had no previous exposure to pain? Behavioral and physiological reactions can indicate a baby is in pain. Are these reactions—which can range from crying to increased heart rate during medical procedures—reflex responses, or do infants consciously feel pain as a sensory, emotional and cognitive experience?

Imaging studies in adults reveal that there is a complex network of brain activity that underlies pain, known as the ‘pain matrix’. Use of a technique called functional magnetic resonance imaging (fMRI)—which measures brain activity by detecting changes in blood oxygenation level—has identified many regions of the brain that are activated during acute pain in adults: these regions include the thalamus, the somatosensory cortex and the amygdala (Denk et al., 2014). However, whether a pain matrix exists in infants has so far received little attention (Williams et al., 2015).

Now, in eLife, Rebeccah Slater and colleagues at the University of Oxford—with Sezgi Goksan as first author—demonstrate for the first time that most (18/20) of the brain regions involved in pain in adults are also activated in healthy full-term newborn babies (Figure 1; Goksan et al., 2015). The most surprising difference was the lack of activation of the amygdala in the babies. This region is involved in emotional responses and develops earlier than other brain regions that are involved in cognition. Furthermore, the finding that the fMRI response in newborn babies occurs at lower sensory thresholds than in adults confirms the heightened sensitivity to pain in newborns that has been reported in previous studies of behavioral responses.

**Figure 1.**
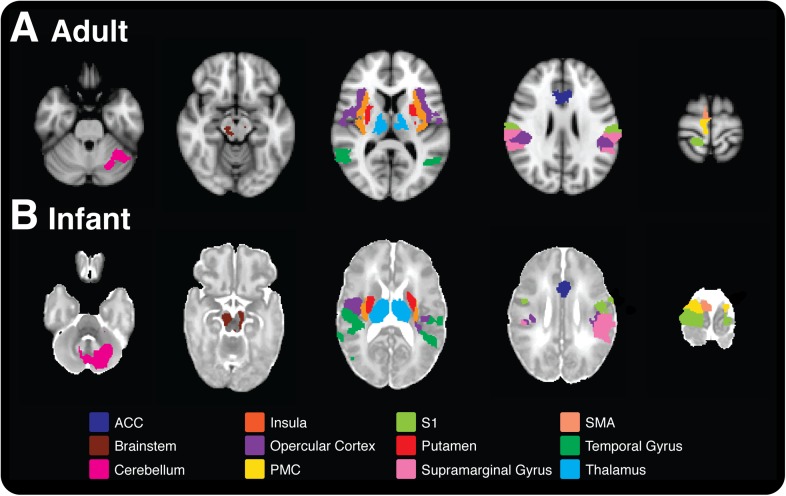
Pain is associated with similar patterns of brain activity in adults and newborn babies. This figure–taken from Goksan et al. (2015)–shows brain activity in selected brain regions in adults (top) and infants (bottom) in response to a prick in the foot that did not break the skin. Each colour represents activity in a different region of the brain; the activity is overlaid onto cross sections of a standard brain template for an adult or newborn baby, with the top-most cross section being on the right. ACC: anterior cingulate cortex, S1: primary somatosensory cortex, PMC: primary motor cortex, SMA: supplementary motor area.

An important question is whether the pain matrix can be used as a universal objective assessment of the pain experienced by individuals. In adults, verbal reports of pain and changes in neural activity are not viewed as the same phenomenon (Robinson et al., 2013). Although activated brain regions correlate with self-reports of pain intensity (Apkarian et al., 2011), both are viewed as providing complementary information because pain experience is not directly reflected in the extent or degree of brain activation.

There has been a major debate for decades as to whether pain is a learned phenomenon (Rodkey and Pillai Riddell, 2013). Goksan et al. lay this issue to rest by showing that no prior experience of pain is necessary to produce sensory and some emotional responses, but the cognitive aspect of pain emerges later in development. Therefore fMRI shows great promise in understanding the ability of the infant brain to process touch and other potentially harmful stimuli.

However, the activity of neurons does not necessarily correspond to the subjective feelings experienced by the individual. Newborn babies have a relatively advanced ‘limbic system’ so are likely to experience some form of emotion, but they are not able to place pain into a cognitive framework in the way that adults can. The fact that Goksan et al. did not observe activity in the orbitofrontal cortex—a region involved in decision-making—in newborn babies is due to the later development of this brain region after birth.

Can fMRI be used to study the vulnerability of premature babies and other critically ill infants to pain? Very premature babies (those born 2 to 4 months early) generally spend weeks to months in neonatal intensive care units in hospitals where they undergo many medical procedures. The infant brain develops rapidly during this time, and extensive exposure to invasive procedures in very premature newborns is associated with altered microstructure and function in the immature brain, even after accounting for how premature the baby was and any health conditions related to their early birth (Brummelte et al., 2012; Zwicker et al., 2013). These adverse effects may be due to changes in blood flow in particular brain regions and changes in neurons caused by overactivation, but it is not clear what mechanisms are involved.

Imaging research will contribute to refining the assessment of pain in infants and potentially may guide clinical practice. Essentially, Goksan et al. do not propose that newborn babies' experience of pain is the same as the adult sensation. Nor do they maintain that this imaging technique should be the new ‘gold standard’ for assessing pain in individuals that cannot speak, or that it represents a more reliable/objective method to evaluate pain. Instead, the work of Goksan et al. provides an important foundation to move forward the study of pain in early life, while being aware of the challenges that come with interpreting functional imaging.
